# Steam Train Sign in Slow Orthostatic Tremor

**DOI:** 10.1002/mdc3.13422

**Published:** 2022-02-17

**Authors:** Bart E.K.S. Swinnen, Thijs Boerée, Anne‐Fleur van Rootselaar

**Affiliations:** ^1^ Department of Neurology and Clinical Neurophysiology Amsterdam University Medical Centers, Amsterdam Neuroscience, University of Amsterdam Amsterdam The Netherlands

The helicopter sign is a pathognomonic sign of orthostatic tremor constituting the auditory correlate of the characteristic high frequency (13 to 18 Hz) tremor in the legs upon standing.^1^ The auditory correlate of low frequency tremor in slow orthostatic tremor is however unknown.

Here we present a 57‐year‐old patient with progressive instability upon standing and to a lesser extent during gait since the age of 50. Clinical examination revealed a stiff, broad‐based and slow gait. Upon standing there was an exaggerated body sway, intermittent knee bending, a broad base of support, and a hem sign (i.e., trembling of the hem of the skirt or long shirt covering the thigh). There was a mild postural tremor in the arms. With auscultation a sound reminiscent of a steam train was noted in the leg muscles upon standing. This was corresponding with a 4 to 5 Hz tremor on surface EMG tremor recording (Video S1). There was a moderate coherence between left and right corresponding leg muscles, and there were no signs supporting a functional etiology. Brain MRI, dopamine transporter imaging, muscle MRI, and needle electromyography were unremarkable. She was diagnosed with slow orthostatic tremor of idiopathic etiology. Symptoms were not responsive to clonazepam, propranolol or levodopa.

**Video 1:** Tremor registration with surface EMG of the limbs upon standing. Visual (sweep duration 8 seconds) and auditory signal of the left anterior tibial muscle demonstrates a 4 to 5 Hz tremor and sound reminiscent of a steam train, respectively. Video content can be viewed at https://onlinelibrary.wiley.com/doi/10.1002/mdc3.13422

This case demonstrates a novel clinical sign which may aid in identifying patients with slow orthostatic tremor in a similar way the helicopter sign being an alert for primary (fast) orthostatic tremor. The sensitivity and specificity of the steam train sign is however still unknown and requires further investigation. Slow orthostatic tremor is a disorder with clinical and pathophysiological overlap with primary (fast) orthostatic tremor. Slow orthostatic tremor is however more often associated with other neurological features like parkinsonism, cerebellar ataxia, dystonia, spasticity or myelopathy, and might even be secondary to another (hereditary) neurological disorder underlying these features.^2^

## Author Roles

1. Patient care; 2. Video editing; 3. Manuscript: A. Writing of the first draft, B. Review and critique

BEKS: 1, 3A

TB: 2, 3B

A‐FvR: 1, 3B

## Disclosures

**Ethical Compliance Statement:** The authors confirm that the approval of an institutional review board was not required for this work. Written informed consent has been obtained from the patient. We confirm that we have read the Journal's position on issues involved in ethical publication and affirm that this work is consistent with those guidelines.

**Funding Sources and Conflicts of Interest**: The authors declare that there are no funding sources or conflicts of interest to report.

**Financial Disclosures for the Previous 12 Months:** The authors declare that there are no disclosures to report.
